# Screening and mutation analysis of phenylalanine hydroxylase deficiency in newborns from Jiangxi province

**DOI:** 10.3389/fgene.2023.1049816

**Published:** 2023-02-09

**Authors:** Baitao Zeng, Qing Lu, Shaohong Chen, Huizhen Guan, Xiaolan Xu, Yongyi Zou, Feng Wang, Shuhui Huang, Yanqiu Liu, Bicheng Yang

**Affiliations:** ^1^ Department of Medical Genetics, Jiangxi Maternal and Child Health Hospital, Nanchang, China; ^2^ Jiangxi Provincial Key Laboratory of Birth Defect for Prevention and Control, Jiangxi Maternal and Child Health Hospital, Nanchang, China

**Keywords:** phenylalanine hydroxylase deficiency, newborn screening, mutational spectrum, arbitrary values, Jiangxi province

## Abstract

**Background:** Phenylalanine hydroxylase deficiency (PAHD) is an autosomal recessive disorder of amino acid metabolism and caused by mutations in the phenylalanine hydroxylase (*PAH*) gene. Without timely and appropriate dietary management, the disturbance of amino acid metabolism may impair cognitive development and neurophysiological function. Newborn screening (NBS) can aid the early diagnosis of PAHD, which can give accurate therapy to PAHD patients in time. In China, the PAHD incidence and *PAH* mutation spectrum vary enormously across the provinces. A total of 5,541,627 newborns from Jiangxi province were screened by NBS between 1997 and 2021.

**Method:** One seventy one newborns from Jiangxi province were diagnosed with PAHD. By Sanger sequencing and the multiplex ligation-dependent probe amplification (MLPA) analysis, mutation analysis was performed in 123 PAHD patients. Using an arbitrary values (AV)-based model, we compared the observed phenotype with the predicted phenotype based on the genotype.

**Results:** In this study, we speculated the PAHD incidence of Jiangxi province was about 30.9 per 1,000,000 live births (171/5,541,627). We summarized the *PAH* mutation spectrum in Jiangxi province for the first time. Two novel variants (c.433G > C, c.706 + 2T > A) were found. The most prevalent variant was c.728G > A (14.1%). The overall prediction rate of the genotype-phenotype was 77.4%.

**Conclusion:** This mutation spectrum is very meaningful to improve the diagnostic rate of PAHD and to increase the accuracy genetic counseling. This study offers data for the genotype-phenotype prediction suitable for Chinese population.

## 1 Introduction

Phenylalanine hydroxylase deficiency (PAHD) is an autosomal recessive disorder of amino acid metabolism and its prevalence is about 62.8 per 1,000,000 live births in China (Hillert et al., 2020). It is caused by mutations in the phenylalanine hydroxylase (*PAH*) gene. To date, the *PAH* Gene Locus-Specific Database (PAHvdb) contains 1584 *PAH* variants in total. The enzymatic inactivity of *PAH* variants inhibits the completion of L-phenylalanine (Phe) to L-tyrosine (Tyr) conversion, causing hyperphenylalaninemia (HPA) (Opladen et al., 2012). In addition, its cofactor tetrahydrobiopterin (BH4) deficiency can also lead to HPA (Ye et al., 2018). Without timely and appropriate intervention, the consequent Phe accumulation may impair cognitive development and neurophysiological function (Wang et al., 2019b; Tendi et al., 2022). Based on the degree of Phe accumulation, PAHD is divided into three groups: classic phenylketonuria (cPKU), mild phenylketonuria (mPKU) and mild HPA (mHPA) (Lin et al., 2022). The dietary management is an effective therapeutic approach for PAHD, but the extent of restriction which each group required is different (MacDonald et al., 2020; Firman et al., 2021; Quinn et al., 2022).

Numerous studies have found that the early diagnosis and treatment is essential for the prognosis of PAHD (Huijbregts et al., 2018; van Spronsen et al., 2021; Dobrowolski et al., 2022). The early diagnosis can provide guidelines for a low-phenylalanine diet of PAHD patients (van Spronsen et al., 2021). Newborn screening (NBS) is an effective and low cost method for the early diagnosis (Poloni et al., 2021). Many provinces of China have used NBS for a very long time (Lin et al., 2022). The result of biochemical testing in NBS can provide the early diagnosis of PAHD, whereas the final diagnostic confirmation needs the help of mutational analysis (Opladen et al., 2020).

The clinical phenotype in PAHD patients is significantly related to the residual enzyme activity of PAH and the relationship between enzyme activity and *PAH* variants has been extensively studied (Blau, 2016; Zhang et al., 2019). Hence, some models have been developed to achieve the genotype-phenotype prediction (Blau, 2016). Practical tests showed that an arbitrary values (AV)-based model was easy to analyze and had a higher predicted rate (Hamilton et al., 2018; Wang et al., 2018). Each *PAH* variant has an AV and the sum of the two AVs from both variants on the *PAH* gene is to predict phenotype (Guldberg et al., 1998). In particular, the AV-based model is of better prediction ability in Chinese PAHD patients (Zhu et al., 2017; Li et al., 2018). In China, geographical location and ethnic composition of provinces are significantly different. Therefore, provinces vary enormously in the PAHD incidence and the *PAH* mutation spectrum (Yan et al., 2019; Tao et al., 2021). Jiangxi province lies in south-central China. To facilitate accurate diagnosis and individualized genetic counseling, it’s necessary to investigate the characteristics of *PAH* gene variants in local populations.

Here, we successfully extracted genomic DNA from whole blood of 123 PAHD patients and their parents. Then, taking advantage of Sanger sequencing and the multiplex ligation-dependent probe amplification (MLPA) analysis, we summarized the *PAH* mutation spectrum of Jiangxi province for the first time. Further, the comparison of the phenotype predicted from genotype with the observed phenotype was performed.

## 2 Materials and methods

### 2.1 Participants

From October 1997 to December 2021, the newborns were recalled to the Neonatal Screening Center of Jiangxi Maternal and Child health Hospital for further diagnosis, due to high blood Phe concentration in newborn screening. Following criteria for diagnosing HPA in China (Yang et al., 2014), the ratio of blood Phe concentration to tyrosine (Phe/Tyr) was greater than two and blood Phe concentration was above 120 μmol/L. BH4D were excluded by analysis of urinary pterins profile, BH4 loading test and determination of dihydropteridine reductase (DHPR) in red blood cells (PAHD had the normality of pterins profile and normality of DHPR, while BH4D had the abnormity of pterins profile and normality of DHPR). In this study, a total of 123 unrelated patients diagnosed with PAHD were recruited. All participants originated from Jiangxi province, including 73 males and 50 females. According to the consensus about the diagnosis and treatment of hyperphenylalaninemia in China (Yang et al., 2014), PAHD patients were divided into three groups: cPKU (Phe ≥ 1200 μmol/L), mPKU (360 μmol/L ≤ Phe ≤ 1200 μmol/L) and mHPA (120 μmol/L ≤ Phe ≤ 360 μmol/L). This study was approved by the Clinical Research Ethics Committees of Jiangxi Maternal and Child health Hospital, Nanchang, China. All parents/legal guardians of the patients were provided for written informed consent.

### 2.2 Molecular—Genetics analysis

Genomic DNA was extracted from peripheral blood samples of patients and their parents using a QIAamp DNA Mini Kit (Qiagen). Next, PCR and DNA sequencing of *PAH* gene were performed to determine the causative variant in each family. Each exon of *PAH* gene was amplified using the forward primer and the reverse primer designed by Primer-BLAST (https://www.ncbi.nlm.nih.gov/tools/primer-blast/) (Supplementary Table S1). The PCR was performed through 2x Taq PCR Master MixII (KT211,TIANGEN) according to the manufacturer’s protocol. Amplification was carried out at 95°C for 5 min for initial denaturing, then 30 cycles at 95°C for 40 s, at 57°C for 30 s and at 72°C for 35 s, followed by a final extension of 8 min at 72°C in a T100 Thermal Cycler for the Classroom (BIO-RAD). Thirteen PCR products were sequenced by a sequencing provider (Tsingke, Changsha). Sequencing results were aligned with the *PAH* transcript (NM_00027) to precisely identify the nucleotide variants by Seqman Pro. The disease-causing mutations recorded in the Human Gene Mutation Database (HGMD, http://www.hgmd.cf.ac.uk/ac/validate.php) or PAHvdb Database (http://www.biopku.org/home/pah.asp) of variants detected by sequencing were selected as the causative variant in each family. Mutation nomenclature was based on the HGVS guidelines (https://www.HGVS.org/varnomen).

When no variant or one variant were detected in *PAH* allele by sequencing, the SALSA MLPA Probemix P055-D1PAH kit (MRC-Holland, Netherlands) were subsequently used to analyze large deletions or duplications, according to the manufacturer’s protocol. Amplification products were separated using ABI 3500DXGenetic analyzer and raw data were analyzed with Coffalyser software. The ratio of the peak area of each *PAH* gene fragment to the corresponding normal controls in the electrophoretograms was calculated to determine the *PAH* copy-number variants. A ratio of 0 was indicative of the presence of homozygous deletion and 0.45–0.65 for heterozygous deletion, 0.80–1.20 for two copies, and 1.30–1.65 for heterozygous duplication.

### 2.3 Phenotypic prediction system

According to Guldberg et al. (1998), an AV model was assigned to each *PAH* variant by referring to the PAHvdb database. The sum of the two AVs from both variants on the *PAH* gene was to predict phenotype. These predicted phenotypes were classified as the three-phenotype (AV sum of cPKU = 2, 2 < AV sum of mPKU < 9 and AV sum of HPA > 8).

### 2.4 Statistical analysis

Graph-Pad Prism version 8.0.1 software was used for generating statistical charts in the study. The *t*-test analysis was applied for differences between groups. Statistical significance was obtained only if the *p*-value was less than 0.05.

## 3 Results

### 3.1 Neonatal PAHD screening

A total of 5,541,627 newborns were screened for neonatal diseases in our center between 1997 and 2021. The screening rate increased from 1.65% (5,391/359,525) in 1997 to 97.68% (339,839/347,910) in 2021 (Table 1). In the past 25 years, 171 newborns were diagnosed as PAHD patients in total. Therefore, we speculated that the prevalence of PAHD in Jiangxi province was about 30.9 per 1,000,000 live births (171/5,541,627). Among 171 PAHD patients, 48 patients diagnosed a long time ago were very difficult to be recalled for further genetic diagnosis and the remaining 123 patients were enrolled to characterize the mutational spectrum (Supplementary Table S2). These patients were comprised of 49 cases of cPKU, 35 cases of mPKU and 39 cases of mHPA (Supplementary Table S2).

**TABLE 1:** Newborn screening data of Jiangxi province from 1997 to 2021.

Year	Number of born alive	Total number of screenings	Screening rate (%)	Number of HPA patients
1997	359,525	5,931	1.65	0
1998	364,584	24,356	6.68	2
1999	364,317	24,869	6.83	2
2000	379,872	27,276	7.18	0
2001	381,421	25,292	6.63	4
2002	377,001	27,399	7.27	2
2003	391,866	26,564	6.78	2
2004	427,598	27,757	6.49	0
2005	466,061	25,473	5.47	1
2006	502,914	56,380	11.21	3
2007	530,240	93,930	17.71	4
2008	599,438	131,522	21.94	8
2009	603,849	158,652	26.27	7
2010	617,942	180,116	29.15	7
2011	631,382	216,750	34.33	6
2012	638,293	298,121	46.71	10
2013	625,075	390,438	62.46	20
2014	648,071	483,290	74.57	15
2015	628,241	510,196	81.21	16
2016	617,909	562,398	91.02	23
2017	606,707	562,154	92.66	18
2018	518,470	489,484	94.41	22
2019	479,567	457,372	95.37	26
2020	409,448	396,068	96.73	25
2021	347,910	339,839	97.68	17

### 3.2 Mutation analysis of sanger sequencing

**FIGURE 1:**
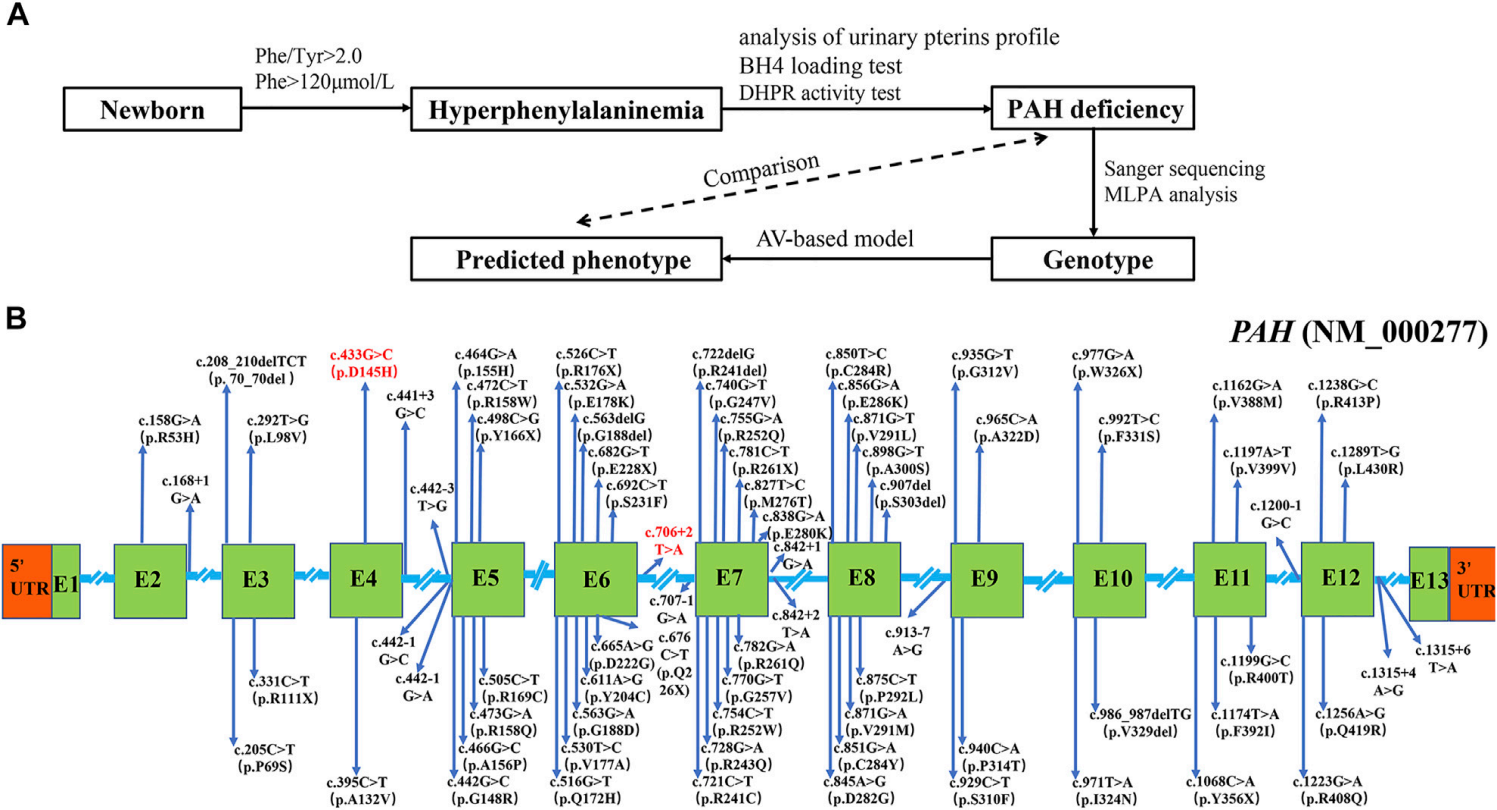
Mutation analysis of 123 PAHD patients. **(A)** Workflow of PAHD patient’s enrollment and follow-up testing in the study. **(B)**The disease-causing mutations were identified by Sanger sequencing in Jiangxi province. Novel mutations were marked red. Abbreviations: E, exon.

We applied Sanger sequencing and MLPA analysis to detect *PAH* variants in 123 enrolled patients, followed by the phenotype prediction based on the genotype in this study (Figure 1A). A total of 233 disease-causing mutations were identified by Sanger sequencing in the 246 alleles of 123 PAHD patients, with a detection rate of 94.7% (233/246) (Supplementary Table S2). These variants were distributed throughout the coding exons and splicing regions of the *PAH* gene, and particularly focused on exon 5–8 (Figure 1B). No variants were in exon 1 and 13 (Figure 1B). Subsequently analyzing parental mutations showed that two disease-causing mutations were determined in both alleles of *PAH* in 111 patients (111/123, 90.2%), 11 patients harbored one mutant allele (11/123, 8.9%) and one patient had no mutation on the *PAH* allele (1/123, 0.8%) (Supplementary Table S2).

### 3.3 Novel mutations

**FIGURE 2:**
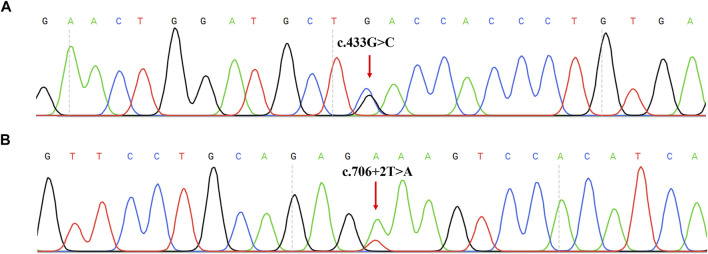
Two novel mutations identified in 123 PAHD patients. **(A)** Sanger sequencing results of the c.433G>C mutation on the *PAH* forward sequence. **(B)** Sanger sequencing results of the c.706 + 2T>A mutation on the *PAH* forward sequence.

Two novel variants were not recorded in HGMD and PAHvdb databases in this study: c.433G > C (p.D145H) and c.706 + 2T > A (IVS6+2T > A) (Figure 2). All novel variants mutations were not present in the population frequency databases. Mutation analysis of probands and their parents showed that c.433G > C and c.706+2T > A were in compound heterozygosity with the known variants (c.442-1G > A and c.721C > T), respectively (Supplementary Table S2).

### 3.4 Large-scale deletion/duplication analysis

**FIGURE 3:**
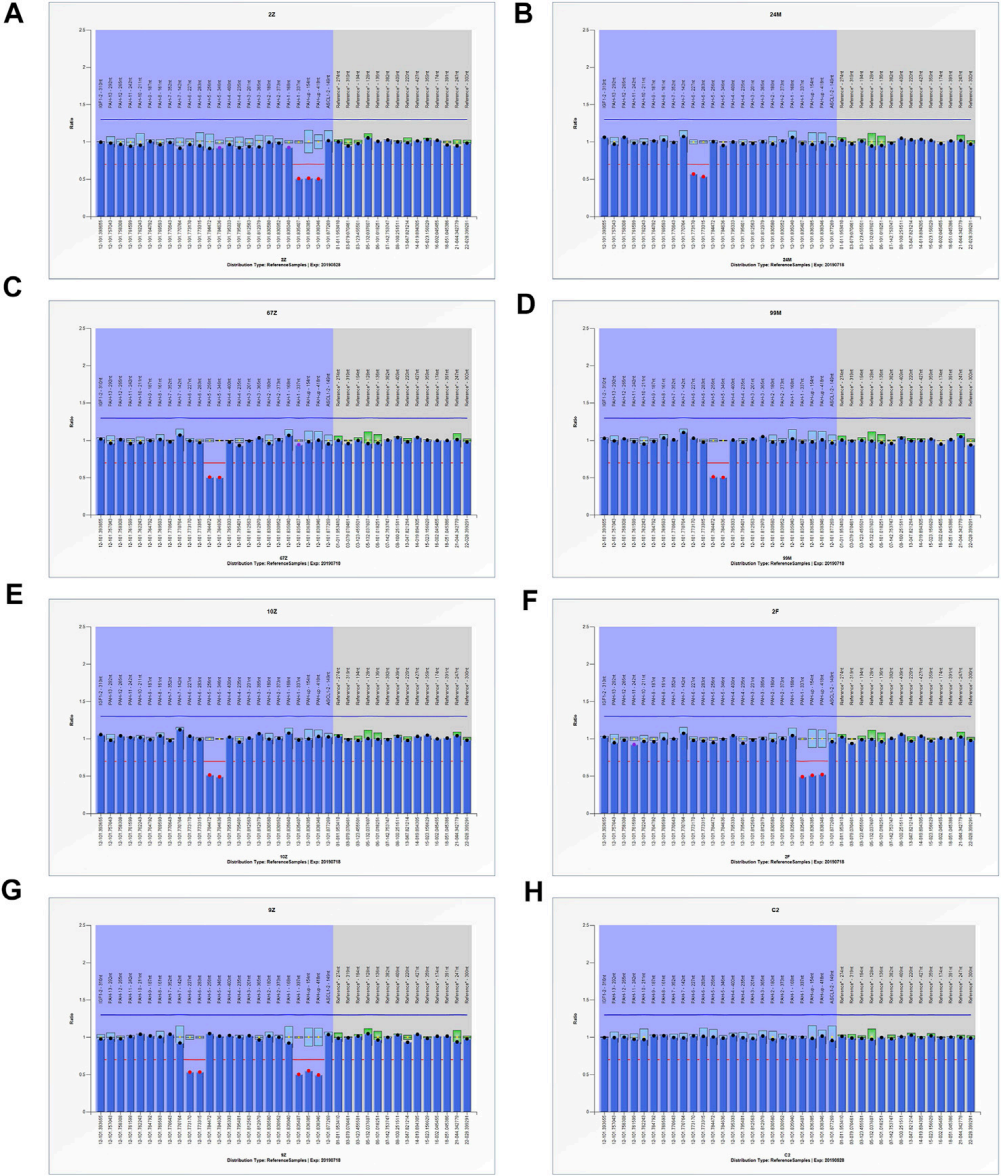
MLPA testing of seven PAHD patients. **(A, F)** The signal for the area spanning the 5′-UTR and Exon1 was nearly half of the signal observed for the normal sample. **(B)** The signal for the *PAH* Exon6 was nearly half of the signal observed for the normal sample. **(C, D, E)** The signal for the *PAH* Exon5 was nearly half of the signal observed for the normal sample. **(G)** Both the signal for the *PAH* Exon6 and the signal for the area spanning the 5′-UTR and Exon1 was nearly half of the signal observed for the normal sample. **(H)** normal sample.

The MLPA analysis was carried out in 12 patients who lacked two disease-causing mutations in both alleles of *PAH*. The large intragenic deletions were detected in seven patients (Figure 3). These results indicated a heterozygous deletion spanning the 5′-UTR and Exon1 in two patients (Family 3A, F), a heterozygous deletion of Exon5 in three patients (Family 3B), a heterozygous deletion of Exon6 in one patient (Family 3C, D, E), a compound heterozygous deletion of Exon5 and 5′-UTR to Exon1 in one patient (Family 3G) (Figure 2).

### 3.5 Mutational analysis of 123 PAHD patients

**FIGURE 4:**
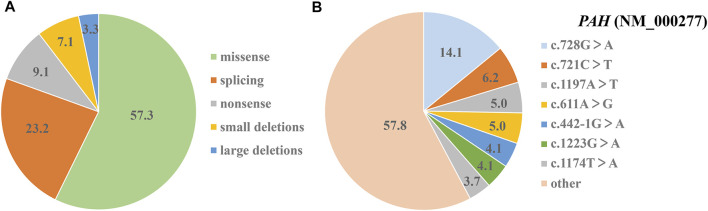
Shares of mutational analysis of 123 PAHD patients. **(A)** The proportion of five different kinds of positive variant findings (%). **(B)** The relative frequency of various detected variants (%).

Sanger sequencing of *PAH* gene showed that 233 variants was positive and MLPA analysis revealed eight large-scale deletions. The combination strategy of Sanger sequencing and MLPA analysis yielded 98.0% positive (241/246) variant findings. Based on mutation types, these variants were grouped in five groups: missense variants (57.3%, 138/241), splicing variants (23.2%, 56/241), non-sense variants (9.1%, 22/241), small deletions (7.1%, 17/241), and large deletions (3.3%, 8/241) (Figure 4A). Among these 241 variants, c.728G > A had the highest frequency (14.1%, 34/241), and other variants, which were just as prevalent, included: c.721C > T (6.2%, 15/241), c.1197A > T (5.0%, 12/241), c.611A > G (5.0%, 12/241), c.442-1G > A (4.1%, 10/241), c.1223G > A (4.1%, 10/241) and c.1174T > A (3.7%, 9/241) (Figure 4B). The seven prevalent variants accounted for 42.3% of all detected variants (Figure 3B).

### 3.6 The comparison of phenotypic prediction with the observed data

The two AVs of 84 enrolled patients were both discovered in the PAHvdb database, whereas the AV was not recorded for one or two *PAH* variants in the 39 remaining cases (Supplementary Table S2). Therefore, 84 patients were enrolled to compare the phenotype predicted with the actual phenotype. The comparison of the predicted phenotype with the observed ones showed that the model had a better prediction ability (65/84, 77.4%), especially in cPKU (39/47, 83.0%) and mHPA patients (21/28, 75.0%) (Table 2). However, the correctly predicted rate of mPKU was only 55.6% (5/9).

**TABLE 2:** The accuracy of phenotype prediction based on the genotype.

AV sum	Phenotype	Predicted number	Observed number			The concordance rate (%)
			cPKU	mPKU	mHPA	
2	cPKU	47	39	6	2	83.0%
2–9	mPKU	9	3	5	1	55.6%
>8	mHPA	28	0	7	21	75.0%
Total		84	42	18	24	77.4%

Abbreviations: AV, sum, the sum of two arbitrary values of the compound heterozygous mutations.

## 4 Discussion

Early detection and treatment play a crucial role in the excellent prognosis, resulting in the increased awareness of NBS (Blau, 2016). The screening rate of neonatal screening was on a constant rise in China and reached to 96.1% at 2016 (Xiang et al., 2019). After 25 years of development, we rose the screening rate to 97.68% from 1.65% in Jiangxi province. The NBS is more and more widely used in Jiangxi province, which indicates that we have achieved the promotion of the NBS.

Screening data suggested that the PAHD incidence differed significantly in each Chinese province, ranging from very low in the southern regions (1/188,679) to high in the northern regions (1/3,492) (Xiang et al., 2019). Our results showed that the incidence in Jiangxi province was about 30.9 per 1,000,000 live births, which was a relatively lower than the average prevalence of 62.8 per 1,000,000 live births in China (Xiang et al., 2019). Likewise, analyzing the newborn screening data, the PAHD incidence of Suzhou and Xiamen of southeastern China (1/9,563 and 1/27,922, respectively) were both higher than the Jiangxi province of south-central China (Wang et al., 2019a; Wang et al., 2019b).

As a result, 233 variants and eight large-scale deletions were found in 246 alleles of 123 PAHD patients and the detection ratio achieved 98.0%. Interestingly, the use of MLPA analysis increased the detection ratio, up from 94.7% to 98.0%. Large deletion and duplication account for 12% and 2.1% of the *PAH* mutations in some ethnic groups (Elhawary et al., 2022). Consistent with previous studies from others, MLPA analysis was a complementary tool to improve the accuracy of diagnosis when no variants were detected in both alleles of *PAH* by sequencing (Lee et al., 2008; Yan et al., 2019; Tao et al., 2021). In particular, we reported here a patient with the compound heterozygous variants of two large-scale deletions. Similar to other provinces (Zhang et al., 2018; Wang et al., 2019a), c.728G > A was also the highest frequency of variant. But the detection rate of 14.1% was lower than 17.7% of northern China and 16.11% of central China (Liu et al., 2017; Zhang et al., 2018). In addition, the highest frequency of *PAH* variant in Hainan province was c.611A > G (Zhao et al., 2020), which was also a common variant in Jiangxi province. There are considerable differences in common variants of different countries and provinces (Liu et al., 2017; Zhou et al., 2022). The low incidence and differences of common variants indicated that the concentrated distribution of *PAH* gene variants in Jiangxi province was less obvious, which was a challenge for genetic counseling. Thus, own spectrum of *PAH* mutations is an efficient tool for appropriate newborn screening and individualized genetic counseling. Other prevalent variants were also present in other provinces (Zhang et al., 2018; Wang et al., 2019a), probably because the population migration broken the region limits.

Some research applied the AV-based model to analyze the relationship between genotype and phenotype in different regions in China (Zhu et al., 2013; Chen et al., 2015; Wang et al., 2018). By contrast, the overall consistency rate of Shanghai area was 54.4% (Zhu et al., 2013), well below the correctly predicted rate of 77.4% in this study. An 84.21% overall consistency rate in north Jiangsu province was higher than that of this study (Wang et al., 2018). Another study found that the consistency rate of Jiangsu province was 38%, which was lower than the data of this study (Chen et al., 2015). AVs of the milder variant are much higher than the severe one, so the PAHD severity depends largely on the milder variant of two *PAH* variants (Li et al., 2018; Elhawary et al., 2022). Nevertheless, we and others discovered that the concordance rate for mPKU was very low in China (Zhu et al., 2013). Since patients (Guldberg et al., 1998) studied were all European, the AVs of some variants would be different in Chinese population (Zhu et al., 2013). For example, previous research by Zhu et al. (2013) showed that AVs of R241C, R243Q, R261Q, V388M, V399V, R408Q, A434D and EX6-96A > G could hardly apply to the PAHD classification of Chinese Han population (Zhu et al., 2013). Our research supported this. After the AVs of R241C changing from 8 to 6, the consistency rate of mPKU and mHPA would rise to 73.3% and 95.5% respectively. It indicated that R241C had particularly pernicious effects on Chinese. The phenotypic difference between different populations might be due to different residual enzymatic activity produced by the ubiquitin–proteasome system (UPS) which can regulate cellular protein turnover (Sarodaya et al., 2022).

In summary, for the first time, we successfully constructed the *PAH* mutation spectrum in Jiangxi province and analyzed the genotypic characteristics of 123 PAHD patients. This mutation spectrum is very meaningful to improve the diagnostic rate of PAHD and to increase the accuracy genetic counseling. We compared the observed phenotype with the predicted phenotype based on the genotype. This study offers data for the genotype-phenotype prediction suitable for Chinese population.

## Data Availability

The original contributions presented in the study are included in the article/Supplementary Material, further inquiries can be directed to the corresponding authors.

## References

[B1] Blau N. (2016). Genetics of phenylketonuria: Then and now. Hum. Mutat. 37, 508–515. 10.1002/humu.22980 26919687

[B2] Chen Y. Jia H. Chen Z. Song J. Liang Y. Pei J. (2015). Mutational spectrum of phenylketonuria in Jiangsu province. Eur. J. Pediatr. 174, 1333–1338. 10.1007/s00431-015-2539-z 25894915

[B3] Dobrowolski S. F. Phua Y. L. Vockley J. Goetzman E. Blair H. C. (2022). Phenylketonuria oxidative stress and energy dysregulation: Emerging pathophysiological elements provide interventional opportunity. Mol. Genet. Metab. 136, 111–117. 10.1016/j.ymgme.2022.03.012 35379539 PMC9832337

[B4] Elhawary N. A. AlJahdali I. A. Abumansour I. S. Elhawary E. N. Gaboon N. Dandini M. (2022). Genetic etiology and clinical challenges of phenylketonuria. Hum. Genomics 16, 22. 10.1186/s40246-022-00398-9 35854334 PMC9295449

[B5] Firman S. Witard O. C. O’Keeffe M. Ramachandran R. (2021). Dietary protein and protein substitute requirements in adults with phenylketonuria: A review of the clinical guidelines. Clin. Nutr. 40, 702–709. 10.1016/j.clnu.2020.11.003 33308842

[B6] Guldberg P. Rey F. Zschocke J. Romano V. François B. Michiels L. (1998). A European multicenter study of phenylalanine hydroxylase deficiency: Classification of 105 mutations and a general system for genotype-based prediction of metabolic phenotype. Am. J. Hum. Genet. 63, 71–79. 10.1086/301920 9634518 PMC1377241

[B7] Hamilton V. Santa María L. Fuenzalida K. Morales P. Desviat L. R. Ugarte M. (2018). Characterization of phenyalanine hydroxylase gene mutations in Chilean PKU patients. JIMD Rep. 42, 71–77. 10.1007/8904_2017_85 29288420 PMC6226402

[B8] Hillert A. Anikster Y. Belanger-Quintana A. Burlina A. Burton B. K. Carducci C. (2020). The genetic landscape and epidemiology of phenylketonuria. Am. J. Hum. Genet. 107, 234–250. 10.1016/j.ajhg.2020.06.006 32668217 PMC7413859

[B9] Huijbregts S. C. J. Bosch A. M. Simons Q. A. Jahja R. Brouwers M. C. G. J. De Sonneville L. M. J. (2018). The impact of metabolic control and tetrahydrobiopterin treatment on health related quality of life of patients with early-treated phenylketonuria: A PKU-cobeso study. Mol. Genet. Metab. 125, 96–103. 10.1016/j.ymgme.2018.07.002 30007854

[B10] Lee Y. W. Lee D. H. Kim N. D. Lee S. T. Ahn J. Y. Choi T. Y. (2008). Mutation analysis of PAH gene and characterization of a recurrent deletion mutation in Korean patients with phenylketonuria. Exp. Mol. Med. 40, 533–540. 10.3858/emm.2008.40.5.533 18985011 PMC2679362

[B11] Li N. He C. Li J. Tao J. Liu Z. Zhang C. (2018). Analysis of the genotype-phenotype correlation in patients with phenylketonuria in mainland China. Sci. Rep. 8, 11251. 10.1038/s41598-018-29640-y 30050108 PMC6062512

[B12] Lin Y. Lin W. Su R. Zheng Z. Fu Q. Wang G. (2022). Newborn screening and genetic features of patients with hyperphenylalaninemia in a southern Chinese population. Clin. Chim. Acta 535, 13–18. 10.1016/j.cca.2022.08.009 35952926

[B13] Liu N. Huang Q. Li Q. Zhao D. Li X. Cui L. (2017). Spectrum of PAH gene variants among a population of Han Chinese patients with phenylketonuria from northern China. BMC Med. Genet. 18, 108. 10.1186/s12881-017-0467-7 28982351 PMC5629770

[B14] MacDonald A. van Wegberg A. M. J. Ahring K. Beblo S. Bélanger-Quintana A. Burlina A. (2020). PKU dietary handbook to accompany PKU guidelines. Orphanet J. Rare Dis. 15, 171. 10.1186/s13023-020-01391-y 32605583 PMC7329487

[B15] Opladen T. Hoffmann G. F. Blau N. (2012). An international survey of patients with tetrahydrobiopterin deficiencies presenting with hyperphenylalaninaemia. J. Inherit. Metab. Dis. 35, 963–973. 10.1007/s10545-012-9506-x 22729819

[B16] Opladen T. López-Laso E. Cortès-Saladelafont E. Pearson T. S. Sivri H. S. Yildiz Y. (2020). Consensus guideline for the diagnosis and treatment of tetrahydrobiopterin (BH4) deficiencies. Orphanet J. Rare Dis. 15, 126. 10.1186/s13023-020-01379-8 32456656 PMC7251883

[B17] Poloni S. dos Santos B. B. Chiesa A. Specola N. Pereyra M. Saborío-Rocafort M. (2021). Current practices and challenges in the diagnosis and management of PKU in Latin America: A multicenter survey. Nutrients 13, 2566. 10.3390/nu13082566 34444728 PMC8399454

[B18] Quinn J. Georgiadis A. Lewis H. B. Jurecki E. (2022). Measuring burden of illness in phenylketonuria (PKU): Development of the PKU symptom severity and impacts scale as a robust patient-reported outcome. Adv. Ther. 39, 971–991. 10.1007/s12325-021-01986-2 34921666 PMC8684342

[B19] Sarodaya N. Tyagi A. Kim H.-J. Kang J.-S. Singh V. Hong S.-H. (2022). Deubiquitinase USP19 extends the residual enzymatic activity of phenylalanine hydroxylase variants. Sci. Rep. 12, 14243. 10.1038/s41598-022-18656-0 35987969 PMC9392723

[B20] Tao Y. Han D. Shen H. Li X. (2021). Spectrum of PAH gene mutations and genotype-phenotype correlation in patients with phenylalanine hydroxylase deficiency from Shanxi province. Brain Dev. 43, 220–229. 10.1016/j.braindev.2020.08.012 32893076

[B21] Tendi E. A. Guarnaccia M. Morello G. Cavallaro S. (2022). The utility of genomic testing for hyperphenylalaninemia. J. Clin. Med. 11, 1061. 10.3390/jcm11041061 35207333 PMC8879487

[B22] van Spronsen F. J. Blau N. Harding C. Burlina A. Longo N. Bosch A. M. (2021). Phenylketonuria. Nat. Rev. Dis. Prim. 7, 36. 10.1038/s41572-021-00267-0 34017006 PMC8591558

[B23] Wang Z.-W. Jiang S.-W. Zhou B.-C. (2018). PAH mutation spectrum and correlation with PKU manifestation in north Jiangsu province population. Kaohsiung J. Med. Sci. 34, 89–94. 10.1016/j.kjms.2017.09.006 29413232 PMC11915670

[B24] Wang T. Ma J. Zhang Q. Gao A. Wang Q. Li H. (2019a). Expanded newborn screening for inborn errors of metabolism by tandem mass spectrometry in Suzhou, China: Disease spectrum, prevalence, genetic characteristics in a Chinese population. Front. Genet. 10, 1052. 10.3389/fgene.2019.01052 31737040 PMC6828960

[B25] Wang X. He Y. Jiang Y. Feng X. Zhang G. Xia Z. (2019b). Screening and mutation analysis of hyperphenylalaninemia in newborns from Xiamen, China. Clin. Chim. Acta 498, 161–166. 10.1016/j.cca.2019.08.021 31445982

[B26] Xiang L. Tao J. Deng K. Li X. Li Q. Yuan X. (2019). Phenylketonuria incidence in China between 2013 and 2017 based on data from the Chinese newborn screening information system: A descriptive study. BMJ Open 9, e031474. 10.1136/bmjopen-2019-031474 PMC6707664 31444193

[B27] Yan Y. Zhang C. Jin X. Zhang Q. Zheng L. Feng X. (2019). Mutation spectrum of PAH gene in phenylketonuria patients in northwest China: Identification of twenty novel variants. Metab. Brain Dis. 34, 733–745. 10.1007/s11011-019-0387-7 30747360

[B28] Yang Y. Ye Y. Subspecial Group of Endocrine Hereditary and Metabolic Diseases Society of Pediatrics Chinese Medical Association Newborn Screening Committee of Professional Society of Birth Defect Prevention and Control Chinese Assocation of Preventive Medical (2014). Consensus about the diagnosis and treatment of hyperphenylalaninemia. Zhonghua Er Ke Za Zhi 52, 420–425. 25190160

[B29] Ye J. Chen C. Yuan Y. Han L. Wang Y. Qiu W. (2018). Haplotype-based noninvasive prenatal diagnosis of hyperphenylalaninemia through targeted sequencing of maternal plasma. Sci. Rep. 8, 161. 10.1038/s41598-017-18358-y 29317692 PMC5760544

[B30] Zhang Z. Gao J.-J. Feng Y. Zhu L.-L. Yan H. Shi X.-F. (2018). Mutational spectrum of the phenylalanine hydroxylase gene in patients with phenylketonuria in the central region of China. Scand. J. Clin. Lab. Invest. 78, 211–218. 10.1080/00365513.2018.1434898 29390883

[B31] Zhang X. Ye J. Shen N. Tao Y. Han L. Qiu W. (2019). *In vitro* residual activities in 20 variants of phenylalanine hydroxylase and genotype-phenotype correlation in phenylketonuria patients. Gene 707, 239–245. 10.1016/j.gene.2019.05.029 31102715

[B32] Zhao Z. Liu X. Huang C. Xu H. Fu C. (2020). Variants of the phenylalanine hydroxylase gene in neonates with phenylketonuria in Hainan, China. Scand. J. Clin. Lab. Invest. 80, 619–622. 10.1080/00365513.2020.1827287 33161754

[B33] Zhou J. Zeng Y. Qiu X. Lin Q. Chen W. Luo J. (2022). Characterization of phenylalanine hydroxylase gene variants and analysis of genotype-phenotype correlation in patients with phenylalanine hydroxylase deficiency from Fujian Province, Southeastern China. Mol. Biol. Rep. 49, 10409–10419. 10.1007/s11033-022-07579-8 36104584 PMC9618490

[B34] Zhu T. Ye J. Han L. Qiu W. Zhang H. Liang L. (2013). Variations in genotype-phenotype correlations in phenylalanine hydroxylase deficiency in Chinese Han population. Gene 529, 80–87. 10.1016/j.gene.2013.07.079 23932990

[B35] Zhu T. Ye J. Han L. Qiu W. Zhang H. Liang L. (2017). The predictive value of genetic analyses in the diagnosis of tetrahydrobiopterin (BH4)-responsiveness in Chinese phenylalanine hydroxylase deficiency patients. Sci. Rep. 7, 6762. 10.1038/s41598-017-06462-y 28754886 PMC5533732

