# Supplementary material for: Screening and mutation analysis of phenylalanine hydroxylase deficiency in newborns from Jiangxi province
Source: Front Genet. 2023 Feb 9;14:1049816. doi: 10.3389/fgene.2023.1049816 (PMC9946975; doi:10.3389/fgene.2023.1049816)
Supplement: Supplementary file 1 [file Table1.DOC]

**Supplementary Information**

Table S1. Primers used in the study.

| Name | Sequence (5’-3’) | Used for the experiment of |
| --- | --- | --- |
| PAH-1-F  PAH-1-R | AGGTGACCTGGAGCATCC  GGATCTCTTTCTCTGGAGGC | PCR for PAH Exon 1 |
| PAH-2-F  PAH-2-R | GAAAGAGTTCATGCTTGCTTTGTCC  GCTCAAATTCAAATCTGCCTGTTCC | PCR for PAH Exon 2 |
| PAH-3-F  PAH-3-R | GTTAGGTTTTCCTGTTCTGG  CTTATGTTGCAAAATTCCTC | PCR for PAH Exon 3 |
| PAH-4-F  PAH-4-R | GTTCTGCCAATCTGTACTCAGG  GTAGAGAAGGTAAGAGGAAGGG | PCR for PAH Exon 4 |
| PAH-5-F  PAH-5-R | GGGAAGGAGACATGCACTGTCATGG  AACTGGATGAGGGCAAGGGAGAAGC | PCR for PAH Exon 5 |
| PAH-6-F | CACAGGTTCTGGTCCCCGAC | PCR for PAH Exon 6 |
| PAH-6-R | CTCTCCTCTGCCTCAATCCTC |
| PAH-7-F | ACTACCAAAGGTCTCCTAGTGC | PCR for PAH Exon 7 |
| PAH-7-R | CAAACCTCATTCTTGCAGCAGG |
| PAH-8-F | TGAGTCTGGCTTGGCTTAAACC | PCR for PAH Exon 8 |
| PAH-8-R | GGTGGGATCATAGAACTGTACC |
| PAH-9-F | TATGTGGGCTGTTCTGAAGG | PCR for PAH Exon 9 |
| PAH-9-R | AGTTTCAAAGACCTGAGGGC |
| PAH-10-F | GTATCCCTTCATCCAGTCAAGG | PCR for PAH Exon 10 |
| PAH-10-R | CCCAGGTTGCATATCAAAACGG |
| PAH-11-F | AAGGAATCGGGGTGAGATGAGAGAAGGGGC | PCR for PAH Exon 11 |
| PAH-11-R | GGTACAAAGTTGCTGTAGACATTGGAGTCC |
| PAH-12-F | ATGCCACTGAGAACTCTCTT | PCR for PAH Exon 12 |
| PAH-12-R | AGTCTTCGATTACTGAGAAA |
| PAH-13-F | GACACTTGAAGAGTTTTTGC | PCR for PAH Exon 13 |
| PAH-13-R | TTTTCGGACTTTTTCTGATG |

**Table S2. Phenotypes and genotypes of 123 PAHD families from Jiangxi,** China.

| **Patient**  **NO** | **Phe**  **(mg/dl)** | **Phenotype** | **Variant** | **Mutation**  **type** | **Source** | **AV** | **Predicted**  **Phenotype**  **based on AV** |
| --- | --- | --- | --- | --- | --- | --- | --- |
| 1 | 11.2 | mPKU | c.611A＞G | Splicing | M | 1 | mHPA |
|  |  |  | c.721C＞T | Missense | P | 8 |  |
| 2 | 20.6 | cPKU | c.728G＞A | Missense | M | 1 |  |
|  |  |  | c.1199G＞C | Missense | P | NR |  |
| 3 | 29.2 | cPKU | c.728G＞A | Missense | M | 1 | cPKU |
|  |  |  | c.728G＞A | Missense | P | 1 |  |
| 4 | 27.7 | cPKU | c.331C＞T | Nonsense | M | 1 | cPKU |
|  |  |  | c.611A＞G | splicing | P | 1 |  |
| 5 | 7.3 | mPKU | c.721C＞T | Missense | M | 8 | mHPA |
|  |  |  | c.728G＞A | Missense | P | 1 |  |
| 6 | 13.2 | mPKU | c.1223G＞A | Missense | P | 4 | mPKU |
|  |  |  | c.728G＞A | Missense | M | 1 |  |
| 7 | 26.2 | cPKU | c.331C＞T | Nonsense | P | 1 | cPKU |
|  |  |  | c.728G＞A | Missense | M | 1 |  |
| 8 | 24.5 | cPKU | c.707-1G＞A | splicing | P | 1 | cPKU |
|  |  |  | c.754C＞T | Missense | M | 1 |  |
| 9 | 9.4 | mPKU | c.728G＞A | Missense | P | 1 |  |
|  |  |  | c.965C＞A | Missense | M | NR |  |
| 10 | 31.0 | cPKU | c.838G＞A | Missense | M | 1 | cPKU |
|  |  |  | c.907_907delT | Deletion | P | 1 |  |
| 11 | 13.2 | mPKU | c.721C＞T | Missense | P | 8 | mHPA |
|  |  |  | c.722delG | Deletion | M | 1 |  |
| 12 | 26.6 | cPKU | c.498C＞G | Nonsense | P | 1 | cPKU |
|  |  |  | c.728G＞A | Missense | M | 1 |  |
| 13 | 29.0 | cPKU | c.1068C＞A | Nonsense | M | 1 |  |
|  |  |  | c.292T＞G | Missense | P | NR |  |
| 14 | 22.6 | cPKU | c.611A＞G | Missense | P | 1 | cPKU |
|  |  |  | c.728G＞A | Missense | M | 1 |  |
| 15 | 4.5 | mHPA | c.721C＞T | Missense | M | 8 | mHPA |
|  |  |  | c.1223G＞A | Missense | P | 4 |  |
| 16 | 22.0 | cPKU | c.755G＞A | Missense | M | 1 | cPKU |
|  |  |  | c.1238G＞C | Missense | P | 1 |  |
| 17 | 26.0 | cPKU | c.728G＞A | Missense | M | 1 | cPKU |
|  |  |  | c.1238G＞C | Missense | P | 1 |  |
| 18 | 20.1 | cPKU | c.907delT | Deletion | M | 1 | cPKU |
|  |  |  | c.1197A＞T | Splicing | P | 1 |  |
| 19 | 20.6 | cPKU | c.498C＞G | Nonsense | M | 1 | mPKU |
|  |  |  | c.1162G＞A | Missense | P | 4 |  |
| 20 | 5.4 | mHPA | c.208_210delTCT | Cds-del | P | 1 | mHPA |
|  |  |  | c.721C＞T | Missense | M | 8 |  |
| 21 | 21.2 | cPKU | c.498C＞G | Nonsense | P | 1 | cPKU |
|  |  |  | c.526C＞T | Nonsense | M | 1 |  |
| 22 | 26.0 | cPKU | c.611A＞G | Missense | M | 1 |  |
|  |  |  | c.850T＞C | Missense | P | NR |  |
| 23 | 25.2 | cPKU | c.331C＞T | Nonsense | P | 1 | cPKU |
|  |  |  | c.842+1G＞A | Splice-5 | M | 1 |  |
| 24 | 24.4 | cPKU | c.1200-1G＞C | Splicing | M | 1 | cPKU |
|  |  |  | c.1200-1G＞C | Splicing | P | 1 |  |
| 25 | 4.5 | mHPA | c.1174T＞A | Missense | P | 8 | mHPA |
|  |  |  | c.1223G＞A | Missense | M | 4 |  |
| 26 | 2.8 | mHPA | c.707-1G＞A | splicing | P | 1 | mHPA |
|  |  |  | c.1174T＞A | Missense | M | 8 |  |
| 27 | 20.6 | cPKU | c.728G＞A | Missense | M | 1 |  |
|  |  |  | c.442G＞C | Missense | P | NR |  |
| 28 | 41.0 | cPKU | c.498C＞G | Nonsense | P | 1 | cPKU |
|  |  |  | c.1197A＞T | Splicing | M | 1 |  |
| 29 | 11.2 | mPKU | c.728G＞A | Missense | M | 1 | cPKU |
|  |  |  | c.1315+6T＞A | Splicing | P | 1 |  |
| 30 | 21.8 | cPKU | c.472C＞T | Missense | P | 1 | cPKU |
|  |  |  | c.728G＞A | Missense | M | 1 |  |
| 31 | 3.3 | mHPA | c.1315+4A＞G | Splicing | M | 1 | cPKU |
|  |  |  | c.1197A＞T | Splicing | P | 1 |  |
| 32 | 22.0 | cPKU | c.611A＞G | Missense | P | 1 | cPKU |
|  |  |  | c.722delG | Deletion | M | 1 |  |
| 33 | 26 | cPKU | c.913-7A＞G | Splicing | P | 1 | cPKU |
|  |  |  | c.929C＞T | Missense | M | 1 |  |
| 34 | 24.4 | cPKU | c.770G＞T | Missense | M | 1 | cPKU |
|  |  |  | c.770G＞T | Missense | P | 1 |  |
| 35 | 5.4 | mHPA | c.442-1G＞C | Splicing | M | NR |  |
|  |  |  | c.1174T＞A | Missense | P | 8 |  |
| 36 | 32 | cPKU | c.728G＞A | Missense | M | 1 | cPKU |
|  |  |  | c.728G＞A | Missense | F | 1 |  |
| 37 | 14.4 | mPKU | c.728G＞A | Missense | P | 1 | mPKU |
|  |  |  | c.1223G＞A | Missense | M | 4 |  |
| 38 | 21.1 | cPKU | c.907delT | Deletion | M | 1 | cPKU |
|  |  |  | c.1197A＞T | Splicing | P | 1 |  |
| 39 | 3.0 | mHPA | c.728G＞A | Missense | P | 1 | mHPA |
|  |  |  | c.464G＞A | Missense | M | 8 |  |
| 40 | 24.6 | cPKU | c.331C＞T | Nonsense | P | 1 | cPKU |
|  |  |  | c.842+1G＞A | Splicing | M | 1 |  |
| 41 | 4.7 | mHPA | c.395C＞T | Missense | P | 8 | mHPA |
|  |  |  | c.563delG | Deletion | M | 1 |  |
| 42 | 28.3 | cPKU | c.442-1G＞A | Splicing | M | 1 | cPKU |
|  |  |  | c.728G＞A | Missense | P | 1 |  |
| 43 | 15.0 | mPKU | c.935G＞T | Missense | M | NR |  |
|  |  |  | c.1223G＞A | Missense | P | 4 |  |
| 44 | 22.7 | cPKU | c.728G＞A | Missense | M | 1 | cPKU |
|  |  |  | c.1197A＞T | Splicing | P | 1 |  |
| 45 | 30.3 | cPKU | c.611A＞G | Splicing | M | 1 | cPKU |
|  |  |  | c.838G＞A | Missense | P | 1 |  |
| 46 | 26.2 | cPKU | c.845A＞G | Missense | M | NR |  |
|  |  |  | c.1238G＞C | Missense | P | 1 |  |
| 47 | 5.3 | mHPA | c.442-1G＞A | Splicing | P | 1 | cPKU |
|  |  |  | c.728G＞A | Missense | M | 1 |  |
| 48 | 14.0 | mPKU | c.563G＞A | Missense | P | 4 |  |
|  |  |  | c.851G＞A | Missense | M | NR |  |
| 49 | 8.7 | mPKU | c.722delG | Deletion | M | 1 | cPKU |
|  |  |  | c.838G＞A | Missense | P | 1 |  |
| 50 | 29.5 | cPKU | c.728G＞A | Missense | M | 1 | cPKU |
|  |  |  | c.1068C＞A | Nonsense | P | 1 |  |
| 51 | 3.8 | mHPA | c.442-1G＞A | Splicing | M | 1 | cPKU |
|  |  |  | c.907delT | Nonsense | P | 1 |  |
| 52 | 33.4 | cPKU | c.442-1G＞A | Splicing | M | 1 | cPKU |
|  |  |  | c.781C＞T | Nonsense | P | 1 |  |
| 53 | 9.5 | mPKU | c.721C＞T | Missense | M | 8 | mHPA |
|  |  |  | c.728G＞A | Missense | P | 1 |  |
| 54 | 45 | cPKU | c.692C＞T | Missense | M | 1 | cPKU |
|  |  |  | c.728G＞A | Missense | P | 1 |  |
| 55 | 10.0 | mPKU | c.1223G＞A | Missense | M | 4 | mPKU |
|  |  |  | c.1223G＞A | Missense | P | 4 |  |
| 56 | 4.7 | mHPA | c.442-1G＞A | Splicing | M | 1 |  |
|  |  |  | c.516G＞T | Missense | P | NR |  |
| 57 | 34.7 | cPKU | c.168+1G＞A | Splicing | P | 1 | cPKU |
|  |  |  | c.611A＞G | Splicing | M | 1 |  |
| 58 | 28.1 | cPKU | c.722delG | Deletion | P | 1 | cPKU |
|  |  |  | c.728G＞A | Missense | M | 1 |  |
| 59 | 15.5 | mPKU | c.728G＞A | Missense | P | 1 |  |
|  |  |  | c.971T＞A | Missense | M | NR |  |
| 60 | 24.6 | cPKU | c.194T＞C | Missense | M | 4 | mPKU |
|  |  |  | c.682G＞T | Nonsense | P | 1 |  |
| 61 | 4.6 | mHPA | c.1197A>T | Splicing | P | 1 | mHPA |
|  |  |  | c.1256A>G | Missense | M | 8 |  |
| 62 | 12.4 | mPKU | c.728G＞A | Missense | M | 1 |  |
|  |  |  | c.1289T＞G | Missense | P | NR |  |
| 63 | 3.8 | mHPA | c.1174T＞A | Missense | M | 8 | mHPA |
|  |  |  | c.1223G＞A | Missense | P | 4 |  |
| 64 | 3.7 | mHPA | c.1174T＞A | Missense | P | 8 | mHPA |
|  |  |  | c.1238G＞C | Missense | M | 1 |  |
| 65 | 9.1 | mPKU | c.442-3T＞G | Splicing | P | NR |  |
|  |  |  | c.611A＞G | Missense | M | 1 |  |
| 66 | 13.3 | mPKU | c.1238G＞C | Missense | P | 1 | cPKU |
|  |  |  | c.498C＞G | Nonsense | M | 1 |  |
| 67 | 8.3 | mPKU | c.331C＞T | Nonsense | P | 1 |  |
|  |  |  | c.871G＞A | Missense | M | NR |  |
| 68 | 4.0 | mHPA | 5’-UTR and Exon 1 deletion | Deletion | P | NR |  |
|  |  |  | c.721C＞T | Missense | M | 8 |  |
| 69 | 10.3 | mPKU | Exon 6 deletion | Deletion | M | NR |  |
|  |  |  | c.871G＞T | Missense | P | NR |  |
| 70 | 33.5 | cPKU | Exon 5 deletion | Deletion | P | NR |  |
|  |  |  | c.986_987delTG | Deletion | M | NR |  |
| 71 | 9.7 | mPKU | c.1315+6T＞A | Splicing | P | 1 |  |
|  |  |  | Exon 5 deletion | Deletion | M | NR |  |
| 72 | 16.3 | mPKU | c.208_210delTCT | Deletion | P | 1 |  |
|  |  |  | Exon 5 deletion | Deletion | M | NR |  |
| 73 | 2.4 | mHPA | c.530T＞C | Missense | P | 4 |  |
|  |  |  | 5’-UTR and Exon 1 deletion | Deletion | M | NR |  |
| 74 | 2.1 | mHPA | c.208_210delTCT | Deletion | M | 1 | mHPA |
|  |  |  | c.1174T＞A | Missense | P | 8 |  |
| 75 | 16.6 | mPKU | 5’-UTR and Exon 1 deletion | Deletion | M | NR |  |
|  |  |  | Exon 6 deletion | Deletion | P | NR |  |
| 76 | 15.0 | mPKU | c.1223G＞A | Missense | P | 4 |  |
|  |  |  | - | - | - | - |  |
| 77 | 22.3 | cPKU | c.442-1G＞A | Splicing | M | 1 |  |
|  |  |  | - | - | - | - |  |
| 78 | 12.4 | mPKU | c.442-1G＞A | Splicing | M | 1 |  |
|  |  |  | - | - | - | - |  |
| 79 | 5.7 | mHPA | c.1223G＞A | Missense | P | 4 |  |
|  |  |  | - | - | - | - |  |
| 80 | 3.5 | mHPA | c.898G＞T | Missense | P | 8 |  |
|  |  |  | - | - | - | - |  |
| 81 | 24.0 | cPKU | c.611A＞G | Missense | M | 1 | cPKU |
|  |  |  | c.707-1G＞A | splicing | P | 1 |  |
| 82 | 10.2 | mPKU | c.466G＞C | Missense | M | NR |  |
|  |  |  | c.740G＞T | Missense | P | 1 |  |
| 83 | 11.3 | mPKU | c.442-1G＞A | Splicing | M | 1 | mPKU |
|  |  |  | c.782G＞A | Missense | P | 4 |  |
| 84 | 11.7 | mPKU | c.721C＞T | Missense | M | 8 | mHPA |
|  |  |  | c.728G＞A | Missense | P | 1 |  |
| 85 | 10.3 | mPKU | c.205C＞T | Missense | M | NR |  |
|  |  |  | c.611A＞G | Missense | P | 1 |  |
| 86 | 20.5 | cPKU | c.875C＞T | Missense | P | NR |  |
|  |  |  | c.1197A＞T | Splicing | M | 1 |  |
| 87 | 26.4 | cPKU | c.442-1G＞A | Splicing | P | 1 | cPKU |
|  |  |  | c.907delT | Nonsense | M | 1 |  |
| 88 | 28.1 | cPKU | c.194T＞C | Missense | M | 4 | mPKU |
|  |  |  | c.611A＞G | Missense | P | 1 |  |
| 89 | 13.9 | mPKU | c.473G＞A | Missense | P | 1 | cPKU |
|  |  |  | c.728G＞A | Missense | M | 1 |  |
| 90 | 14.6 | mPKU | c.441+3G＞C | Splicing | M | 1 | cPKU |
|  |  |  | c.441+3G＞C | Splicing | P | 1 |  |
| 91 | 7.1 | mPKU | c.721C＞T | Missense | M | 8 | mHPA |
|  |  |  | c.721C＞T | Missense | P | 8 |  |
| 92 | 43 | cPKU | c.838G＞A | Missense | M | 1 | cPKU |
|  |  |  | c.842+1G＞A | Splicing | P | 1 |  |
| 93 | 14.8 | mPKU | c.208_210delTCT | Cds-del | P | 1 | cPKU |
|  |  |  | c.842+1G＞A | Splicing | M | 1 |  |
| 94 | 28.3 | cPKU | c.907delT | Nonsense | M | 1 | cPKU |
|  |  |  | c.935G＞T | Missense | P | 1 |  |
| 95 | 39.3 | cPKU | c.842+1G＞A | Splicing | P | 1 | cPKU |
|  |  |  | c.1200-1G＞C | Splicing | M | 1 |  |
| 96 | 3.2 | mHPA | c.498C＞G | Nonsense | P | 1 |  |
|  |  |  | c.940C＞A | Missense | M | NR |  |
| 97 | 22.0 | cPKU | c.781C＞T | Nonsense | P | 1 | cPKU |
|  |  |  | c.1197A＞T | Splicing | M | 1 |  |
| 98 | 2.3 | mHPA | c.728G＞A | Missense | P | 1 | mHPA |
|  |  |  | c.827T＞C | Missense | M | 8 |  |
| 99 | 8.5 | mPKU | c.721C＞T | Missense | M | 8 | mHPA |
|  |  |  | c.728G＞A | Missense | P | 1 |  |
| 100 | 10.2 | mPKU | c.706+2T＞A | Splicing | M | NR |  |
|  |  |  | c.721C＞T | Missense | P | 8 |  |
| 101 | 4.3 | mHPA | c.770G＞T | Missense | P | 1 | mHPA |
|  |  |  | c.1174T＞A | Missense | M | 8 |  |
| 102 | 2.4 | mHPA | c.498C＞G | Nonsense | P | 1 | mHPA |
|  |  |  | c.464G＞A | Missense | M | 8 |  |
| 103 | 2.3 | mHPA | c.464G＞A | Missense | M | 8 | mHPA |
|  |  |  | c.464G＞A | Missense | P | 8 |  |
| 104 | 4.9 | mHPA | c.1174T＞A | Missense | M | 8 | mHPA |
|  |  |  | c.1197A＞T | Splicing | P | 1 |  |
| 105 | 2.4 | mHPA | c.158G＞A | Missense | M | 8 | mHPA |
|  |  |  | c.755G＞A | Missense | P | 1 |  |
| 106 | 26.6 | cPKU | c.331C＞T | Nonsense | M | 1 | cPKU |
|  |  |  | c.1197A＞T | Splicing | P | 1 |  |
| 107 | 2.0 | mHPA | c.433G＞C | Missense | M | NR |  |
|  |  |  | c.442-1G＞A | Splicing | P | 1 |  |
| 108 | 16.6 | mPKU | c.782G＞A | Missense | M | 4 | mPKU |
|  |  |  | c.856G＞A | Missense | P | 1 |  |
| 109 | 37.3 | cPKU | c.331C＞T | Nonsense | P | 1 | cPKU |
|  |  |  | c.907_907delT | Deletion | M | 1 |  |
| 110 | 2.4 | mHPA | c.158G＞A | Missense | P | 8 | mHPA |
|  |  |  | c.1197A＞T | Splicing | M | 1 |  |
| 111 | 2.6 | mHPA | c.464G＞A | Missense | P | 8 | mHPA |
|  |  |  | c.1197A＞T | Splicing | M | 1 |  |
| 112 | 25.0 | cPKU | c.728G＞A | Missense | P | 1 | cPKU |
|  |  |  | c.977G＞A | Nonsense | M | 1 |  |
| 113 | 2.3 | mHPA | c.158G＞A | Missense | P | 8 | mHPA |
|  |  |  | c.728G＞A | Missense | M | 1 |  |
| 114 | 2.2 | mHPA | c.505C＞T | Missense | M | NR |  |
|  |  |  | c.532G＞A | Missense | P | NR |  |
| 115 | 3.3 | mHPA | c.721C＞T | Missense | M | 8 |  |
|  |  |  | c.940C＞A | Missense | P | NR |  |
| 116 | 2.6 | mHPA | c.158G＞A | Missense | P | 8 |  |
|  |  |  | c.842+2 T＞A | Splicing | M | NR |  |
| 117 | 2.6 | mHPA | c.158G＞A | Missense | M | 8 | mHPA |
|  |  |  | c.611A＞G | Missense | P | 1 |  |
| 118 | 3.6 | mHPA | c.158G＞A | Missense | P | 8 | mHPA |
|  |  |  | c.728G＞A | Missense | M | 1 |  |
| 119 | 5.7 | mHPA | c.721C＞T | Missense | P | 8 | mHPA |
|  |  |  | c.721C＞T | Missense | M | 8 |  |
| 120 | 4.6 | mHPA | c.301G＞A | Missense | P | NR |  |
|  |  |  | c.1238G＞C | Missense | M | 1 |  |
| 121 | 2.3 | mHPA | c.158G＞A | Missense | P | 8 |  |
|  |  |  | c.676C＞T | Missense | M | NR |  |
| 122 | 3.0 | mHPA | c.158G＞A | Missense | M | 8 | mHPA |
|  |  |  | c.728G＞A | Missense | P | 1 |  |
| 123 | 2.5 | mHPA | c.665 A＞G | Missense | M | NR |  |
|  |  |  | c.1174T＞A | Missense | P | 8 |  |

cPKU: classic PKU; mPKU: moderate PKU; mHPA: mild hyperphenylalaninemia; P: Paternal; M: Maternal; AV: Assigned Value; NR: No Record; -: not detected.
